# Medical Association of Georgia

**Published:** 1878-05-20

**Authors:** 


### MEDICAL ASSOCIATION OF GEORGIA.

---

The Medical Association of Georgia held its annual meeting in Atlanta commencing on the 17th of April, and was in session three days.

The Sunday-school Convention which was in session at the same time, diverted the attention of the public so that very few spectators witnessed the proceedings of the society. Nor did there seem to be the usual degree of interest in the work of the association by the members present or the profession generally.

The Association met in the Senate Chamber, and was opened with prayer by the Rev. Mr. Martin of the 1st Presbyterian church

The address of welcome was made by Dr. Jno. M. Johnson in eloquent language. and appropriately responded to by Dr. Charters, of Savannah.

Dr. Battey, the outgoing president, then introduced the president elected at the last meeting, Wm. O'Daniel, who delivered an interesting inaugural address.

About 80 members enrolled their names.

Reports of sections were called for. Many failed to report.

Dr. G. J. Grimes read a paper on tubercular meningitis.

Dr. Greggs made a verbal report of a number of cases in gynæcology.

Dr. Kennon—a paper on fistula in ano.

Dr. A. Mathas—a case of abdominal dropsy, tapped 90 times.

Dr. Love reported a case of menstrual derangement; also a paper on the diagnostic value of the soft palate as compared with the tongue.

Dr. LeHardy—a paper on yellow fever.

Dr. Baird—a paper on moslem therepeutics.

Dr. Gaither—a case of puerperal elampsia.

Dr. A. W. Calhoun—one hundred and five operations for strabismus.

Blood letting—by Dr. Chas. Rauschenburg.

Non-syphiletic corryza—by Dr. Stout.

The true physician—by Dr. T. S. Powell.

Diseases of the uterus—by Dr. Taliaferro.

Sacharated medicines—by Dr. Love.

Corn stalk as material for uterine tents—by Dr. W. T. Goldsmith.

Veratia ointment for neuralgia—by Dr. Stout.

Address by Dr. A. Means on electricity.

On the second day, voluntary papers were called for.

A case of sloughing of the uterus was reported by Dr. Drake.

Obstinate case of hiccough—by Dr. J. B. Roberts.

Gun-shot wound of the spine—by Dr. J. G. Thomas.

Dr. W. R. Burgess, of Macon, orator elect, delivered an interesting address on "Hasty, unwise and unfortunate medical literature."

Dr. H. Smith, of Augusta, read a memorial that had been prepared for presentation to the legislature, petitioning the removal of the special tax on physicians.

The memorial was adopted, and a committee appointed to look after the matter.

Dr. W. Westmoreland made a verbal report on congenital phimosis, in which cases were reported, showing that paralysis of the lower extremities in children not unfrequently results from phimosis.

Dr. C. B. Leitner, of Columbus, presented a paper on tar bandages.

On the third day, a resolution was introduced by Dr. Drake to appoint a committee to report upon the propriety of changing the by-laws so as to dispense with a nominating committee and elect officers by ballot. The resolution elcited a warm discussion, and was ably defended by Drs. J. M. Johnson, H. V. M. Miller, Dr. T. S. Powell and others. Dr. Roach referred to certain action which had transpired in the room of the nominating committee, which smacked of injustice. This elicited an indignant rejoinder from Dr. Baird.

Dr. Roach stated that he intended nothing offensive, he had received his information from Dr. G. S. Crawford, a member of the committee from Fulton.

Dr. Crawford said he would state what did occur and members could form their own conclusions. He had submitted a proposition to the commitiee calculated to harmonize the profession in Atlanta, which proposition was rejected, and the nomination was precipitated in a manner that seemed to him very much like "it was cut and dried."

After some further discussion the resolution was carried, and a committee of 7 was appointed by a vote of 56 to 1, showing an overwhelming opposition to the caucus method of nominating. This committee, however, finding that the nominations were open to ratification or rejection by the body, reported against any change of the present method as unnecessary. Under these circumstances, a majority of the nominations made being satisfactory, the report of the committee was accepted.

The committee on nominations then reported the following names for officers for the ensuing year, which was agreed to:

President—John Thad Johnson, Atlanta.

1st Vice President—W. F. Holt, Macon.

3d Vice President—T. H. Kennon, Milledgeville.

Secretary—Joseph B. Baird, Atlanta.

Treasurer—W. R. Burgess, Macon.

Orator—E. H. Richardson, Cedartown.

Dr. Robert Battey was elected to fill vacancy on board of censors.

The following were appoiuted to report on sections:

FIRST DISTRICT.

Practice—J. G. Thomas, Wm. Duncan and John D. Martin. Surgery—R. P. Myers, G. A. Stone and J. W. Norton. Gynaecology—J. B. Read, J. C. LeHardy and J. P. S. Houston.

SECOND DISTRICT.

Practice—W. M. Bruced, R. L. Hillmen and W. W. Twitty. Surgery—B. R. Dostor, W. A. Strother and T. S. Hopkins. Gynaecology—W. B. Fackett, E. W. Alfriend and T. A. Chappell.

THIRD DISTRICT.

Practice—A. R. Taylor, A. W. Reese and S. B. Hawkins. Surgery—F. M. Jordan, Geo. F. Cooper and A. A. Smith. Gynaecology—F. F. Walker, J. W. Tucker and J. B. Hinkle.

FOURTH DISTRICT.

Practice—A. W. Griggs, D. W. Johnson and P. M. Tidwell. Surgery—G. J. Grimes, W. W. Fitts and John F. Slaughter Gynaecology—J. W. Griggs, T. A. Sanford and F. L. Wisdom.

FIFTH DISTICT.

Practice—J. B. Baird, Paul Faver and J. G. Westmoreland. Surgery—A. W. Calhoun, T. L. Lallerstadt and J. A. McKown. Gynæcology—J. P. Logan, K. P. Moore and T. M. Darnell.

SIXTH DISTRICT.

Practice—Wm. F. Holt, Henry Garther and H. V. Johnson. Surgery—J. L. Harris, B. Hendricks and S. L. Richardson. Gynæcology—W. O'Daniel, W. R. Burgess and J. E. Blackshear.

SEVENTH DISTRICT.

Practic—A. L. Fowler, J. B. S. Holmes and E: H. Richardson. Surgery—R. F. Wright, W. S. Hendricks and W. B. Wells. Gynæcology—R. Battey, C. B. Gordon and T. R. Calhoun.

EIGHTH DISTRICT.

Practice—W. H. Doughty, W. W. Battey and W. H. Foster. Surgery—A. S. Campbell, D. Ford and E. A. Dugas. Gynæcology—H. F. Campbell, J. S. Coleman and R. C. Eve.

NINTH DISTRICT.

Practice—W. T. Hollinsworth, I. H. Goss and J. S. Simmons. Surgery—L. G. Hardeman, A. A. Bell and R. M. Smith. Gynæcology—C. W. Long, J. W. Bailey and J. F. Wersen.

Committee on Publications—J. B. Baird, W. R. Burgess, W. A. Love, W. S. Armstrong and V. H. Taliaferro.

Committee on Necrology—R. P. Myers, H. V. Johnson, C. H. Hall, W. A. Love and A. S. Campbell.

A committee consisting of Drs. Taliaferro, Battey and Powell was appointed to take the communication of Judge Bigham under consideration in regard to lunatics.

There was some discussion on the State Board of Health. The committee appointed at last meeting to advocate and bring the matter before the State Legislature, was continued, though not without indications of opposition. Dr. Stout taking ground that the people were not ready for the measure, in a speech of considerable force and energy, which was warmly applauded.

A lengthy discussion on the subject of uterine tents was indulged in by Drs. Love, Taliaferro and Powell, after the reading of a paper by Dr. Goldsmith.

A number of admissions to membership were made, and many resignations. Among those were Drs. Geo. M. McDowell, H. Perdue, J. E. Cook, B. F. Chambliss, B. F. Rudisill, T. J. Collier, J. C. Blackburn, Fitzgerald, of Macon, and J. J. Knott, of Atlanta.

The initiation fee was continued at $5. Regular dues, $2 per annum for members who attend. Those who do not attend to pay $1.

During the session a banquet was given to the members by the Atlanta physicians, at which wine and fat things were abundant, and many toasts and happy responses were given.

An invitation was extended by Dr. J. M. Johnson of the city to the members to visit his home, which was accepted, and a fine supper and a good, jovial time enjoyed.

A special invitation was also extended to the society to visit the wholesale drug house of Messrs. Hunt, Rankin & Lamar, at which samples of champagne and other articles in the drug line were tested and examined.

They were also invited to a brilliant reception at the Governor's mansion, and to other places of attraction and interest.

The next meeting of the association will take place in Rome, Georgia, on the third Wednesday in April, 1879.

